# CT-based muscle and adipose measurements predict prognosis in patients with digestive system malignancy

**DOI:** 10.1038/s41598-024-63806-1

**Published:** 2024-06-06

**Authors:** Kaiwen Zheng, Xiangliang Liu, Yuguang Li, Jiuwei Cui, Wei Li

**Affiliations:** 1 Cancer Center, The First Hospital of Jilin University, Xinmin St No 126, Changchun, 130021 Jilin China; 2 College of Instrumentation and Electrical Engineering, Jilin University, Changchun, Jilin China

**Keywords:** Body composition analysis, Digestive system cancer, Skeletal muscle, Adipose tissue, Computed tomography, Cancer, Biomarkers

## Abstract

The role of skeletal muscle and adipose tissue in the progression of cancer has been gradually discussed, but it needs further exploration. The objective of this study was to provide an in-depth analysis of skeletal muscle and fat in digestive malignancies and to construct novel predictors for clinical management. This is a retrospective study that includes data from Cancer Center, the First Hospital of Jilin University. Basic characteristic information was analyzed by T tests. Correlation matrices were drawn to explore the relationship between CT-related indicators and other indicators. Cox risk regression analyses were performed to analyze the association between the overall survivals (OS) and various types of indicators. A new indicator body composition score (BCS) was then created and a time-dependent receiver operating characteristic curve was plotted to analyze the efficacy of the BCS. Finally, a nomogram was produced to develop a scored-CT system based on BCS and other indicators. C-index and calibration curve analyses were performed to validate the predictive accuracy of the scored-CT system. A total of 575 participants were enrolled in the study. Cox risk regression model revealed that VFD, L3 SMI and VFA/SFA were associated with prognosis of cancer patients. After adjustment, BCS index based on CT was significantly associated with prognosis, both in all study population and in subgroup analysis according to tumor types (all study population: HR 2.036, *P* < 0.001; colorectal cancer: HR 2.693, *P* < 0.001; hepatocellular carcinoma: HR 4.863, *P* < 0.001; esophageal cancer: HR 4.431, *P* = 0.008; pancreatic cancer: HR 1.905, *P* = 0.016; biliary system malignancies: HR 23.829, *P* = 0.035). The scored-CT system was constructed according to tumor type, stage, KPS, PG-SGA and BCS index, and it was of great predictive validity. This study identified VFD, L3 SMI and VFA/SFA associated with digestive malignancies outcomes. BCS was created and the scored-CT system was established to predict the OS of cancer patients.

## Introduction

Cancer remains one of the leading threats to human health, with an estimated 19.3 million new cancer cases in 2020, according to the International Agency for Research on Cancer. Among them, there were nearly 6 million cases of digestive system malignancies, which accounted for more than 30% of the total number of new cancer cases worldwide^1^. Genetic changes are only one part of the development of digestive system malignancies, while lifestyle and dietary habits may have a more significant impact on them, among which, excessive nutrition and obesity lead to many cancers, including esophageal, colorectal, and gastric cancers^2,3^.

Higher body mass index (BMI) is thought to be associated with more aggressive tumor cells and poorer clinical outcomes for cancer patients, but such claims have been contradicted in multiple studies^4–6^. BMI cannot identify the exact body components that affect health risks, in which case, it cannot directly distinguish between muscle tissue and adipose tissue, nor can it accurately evaluate the distribution of each component, which leads to the inability to identify the hidden obesity of the patients and cannot effectively reflect the real condition of the body. In this context, a series of body composition analysis methods, such as bioelectrical impedance analysis (BIA), are gaining popularity. Cancer patients are in a state of chronic depletion and their body composition is quietly changing. Accurate assessment of a patient's body composition can provide doctors with guidance for individualized and precise treatment. Highly sensitive imaging tools, such as computed tomography (CT) have been recommended to assess muscle quality. It is considered to be the gold standard for evaluating the composition of the body, and by detecting potential changes in the body, it could guide medical decision-makers for timely changes in therapeutic management strategies^7^.

It is feasible to evaluate the response to antitumor therapy and the prognosis of cancer patients using muscle and fat status^8–11^. However, current research is often one-sided, with researchers often exploring the role of only one of these components in depth, ignoring the combined effects of both skeletal muscle and fat. The clinical outcome of patients is the result of a combination of factors, and it is necessary to clarify the specific values of various components considering the important role of body components in cancer patients. The objective of this study was to explore the prognostic value of CT-measured fat and skeletal muscle components in patients with digestive system malignancies and to construct a new indicator for clinical guidance.

## Participants and methods

### Study participants

**Figure 1:**
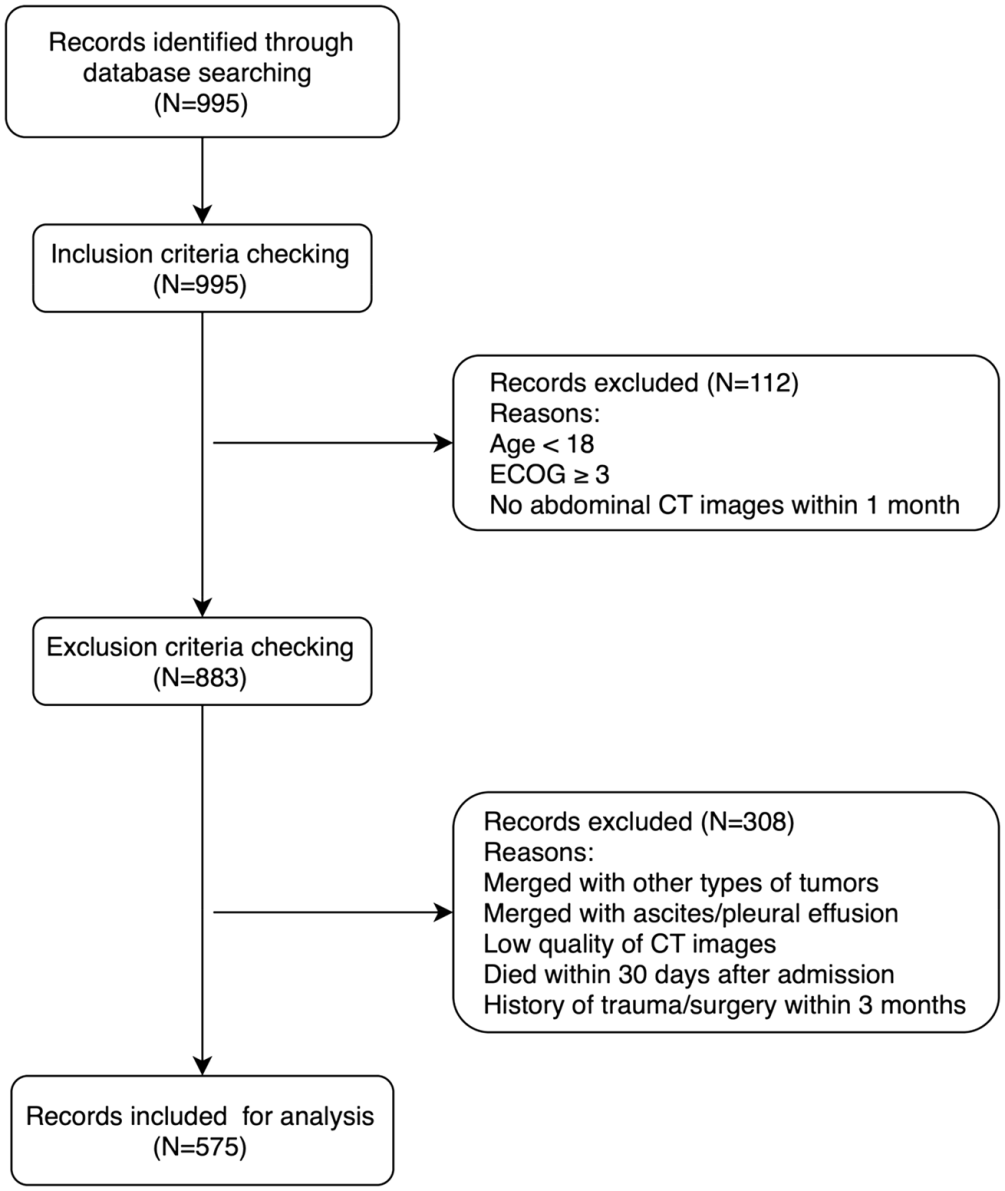
The flow diagram of participants enrolled. ECOG, Eastern Cooperative Oncology Group performance status.

This is a retrospective study that screened patients who were admitted to the Cancer Center of the First Hospital of Jilin University from January 1st, 2015 to December 30th, 2021. The inclusion criteria were as follows: (1) Cancer confirmed by pathology with an Eastern Cooperative Oncology Group performance status of 0, 1, or 2; (2) No anti-tumor therapy or related therapy before assessment; (3) No metabolic disease or autoimmune disease; (4) Abdominal CT examination within 1 month before or after the diagnosis of cancer; (5) Age > 18 years old. Meanwhile, the exclusion criteria were as follows: (1) Patients with other types of tumors; (2) History of trauma or surgery within 3 months; (3) Low-quality CT images; (4) Combined with abdominal effusion/pleural effusion; (5) Receive regular hemodialysis; (6) Died within 30 days of admission. The flowchart of study subject inclusion is shown in Fig. 1.

All pathological staging was defined according to the 8th edition of the American Joint Committee on Cancer (AJCC) TNM staging system. The study was approved by the Ethics Committee of the first affiliated hospital of Jilin University (2017-362), and all data in this research was approved by this committee. This study has been performed in accordance with the Declaration of Helsinki and all study participants have filled out written informed consent for participation.

### Data collection

The Patient-Generated Subjective Global Assessment (PG-SGA) and Karnofsky Performance Status (KPS) questionnaire were completed within 24 h after admission with professional assistance. According to the PG-SGA score, the nutritional status of patients was assessed. We defined well-nourished as PG-SGA ≤ 2, moderate malnutrition as 3 ≤ PG-SGA ≤ 8, and severe malnutrition as PG-SGA ≥ 9^12^. We used the European Organization for Research and Treatment of Cancer’s Quality of Life Questionnaire Core 30 to assess health-related quality of life (HRQoL)^13^.

Basic clinical information was recorded, including sex, age, tumor type, tumor stage, smoking history, alcohol history, and previous nutritional therapy. Serological tests were performed in the early morning of the second day after admission, and relevant serological parameters were recorded, including albumin (Alb), C-reactive protein (CRP), neutrophil count, platelet count, lymphocyte count, serum triglycerides (TG), serum total cholesterol (TC), serum high-density lipoprotein cholesterol (HDL-C) and serum low-density lipoprotein cholesterol (LDL-C). Neutrophil to lymphocyte ratio (NLR), platelet to lymphocyte ratio (PLR) and Onodera's prognostic nutritional index (OPNI) were calculated according to the formula: NLR = Neutrophil count (× 10^9^/L)/lymphocyte count (× 10^9^/L); PLR: Platelet count (× 10^9^/L)/lymphocyte count (× 10^9^/L); OPNI = serum albumin level (g/L) + 5 × total number of lymphocytes in peripheral blood (× 10^9^/L)^14^.

### Anthropometric and BIA examinations

Anthropometric and BIA tests were performed within 24 h after admission with professional assistance. In this study, resistance and capacitance were directly measured in ohms at 50 kHz and 800 mA with an Inbody S10 (Biospace Co). Before the measurement, patients voided their bladders, fasted for at least 2 h, and wore a uniform hospital gown; they also avoided physical activity and remained quiet. Patients' relevant indexes were recorded, including height, weight, circumference of the mid-upper arm (MAC), mid-upper arm muscle circumference (MAMC), skinfold thickness (TSF), and the grip strength of the non-dominant hand (HGS). Height and weight were measured to an accuracy of 1 cm and 0.1 kg, respectively. BMI was calculated according to the formula weight (kg)/height^2^ (m)^2^. Record appendicular skeletal muscle mass (ASM), extracellular water (ECW) and total body water (TBW). Appendicular skeletal muscle mass index (ASMI) = ASM (kg)/height^2^ (m^2^).

### Body composition analysis by CT

We quantified data from cross-sectional unenhanced CT scans used to diagnose tumors or tumor staging. All CT scans were exported by radiologists and engineers of the First Hospital of Jilin University and quality control was performed. The final CT images that met the quality control requirements were quantified and analyzed by sliceOmatic (version 5.0; Tomovision, Montreal, Canada) imaging analysis software.

Cross-sectional CT images at the level of the third lumbar (L3) vertebrae for each patient were analyzed. The third lumbar vertebra was set as a landmark, and two consecutive slices were selected to measure the cross-sectional areas of the skeletal muscle. The mean value of two consecutive images was computed for each patient.

Abdominal fat tissue mainly includes subcutaneous fat and visceral fat. Muscle areas included the psoas, erector spinae, quadratus lumborum, transversus abdominis, external and internal obliques, and rectus abdominis muscles. We quantified the cross-sectional areas of subcutaneous fat area (SFA, cm^2^), visceral fat area (VFA, cm^2^) and skeletal muscle area of the third lumbar vertebrae (L3 SMA) using standard Hounsfield units (HUs). According to previous research results^15,16^, we considered a threshold of -190 to -30 HU as subcutaneous fat, -150 to -50 HU as visceral fat and − 29 to 150 HU as skeletal muscle. Total fat area (TFA, cm^2^) was determined as the sum of the SFA and VFA. In addition, subcutaneous fat density (SFD, HUs), visceral fat density (VFD, HUs) and skeletal muscle density of third lumbar vertebrae (L3 SMD, HUs) were also calculated by the mean attenuation of subcutaneous fat, visceral fat and skeletal muscle using the same CT images, respectively. The ratio of VFA/SFA and VFD/SFD were also recorded. Skeletal muscle index of third lumbar vertebrae (L3 SMI) was calculated according to the formula L3 SMA (cm^2^)/ height^2^ (m^2^). SFA, VFA and TFA were normalized for height^2^ and reported as SFA index (SFAI, cm^2^/m^2^), VFA index (VFAI, cm^2^/m^2^) and TFA index (TFAI, cm^2^/m^2^)^17^.

### X-tile software to determine the optimal cut-off value

In this study, we used X-tile, a software developed by researchers at Yale University to calculate optimal cut-off values for relevant biological information based on outcomes, to establish appropriate cut-off values for the metrics of interest^18^. X-tile plots are created by dividing marker data into two populations: low and high. All possible divisions of the marker data are assessed.

### Follow-up

All patients were treated with the standard treatment protocol recommended by the guidelines according to tumor type and stage after diagnosis. Participants were regularly followed up by trained persons through telephone interviews or outpatient visits to collect data on survival status and time of events. In this study, we took overall survivals (OS) as the endpoint. Participants were followed from the initial admission until they died or until the end of December 2021.

### Statistical analysis

SPSS 26.0 statistical software and R Project for Statistical Computing (version 4.0.5) were used for data analyses. Continuous variables were expressed as the mean ± standard deviation and categorical variables were presented as counts (%). Basic characteristic information of males and females were analyzed by T-tests. The relationship between basic clinical information, anthropometric indicators, serological indicators, BIA data, and CT-related indicators of patients was analyzed using correlation matrices. The optimal cut-off values of CT-related indicators were determined in male and female patients separately using X-tile software and divided into high and low value groups based on the cut-off values. A univariate Cox risk regression model was used to analyze the relationship between OS and CT-related indicators. All statistically significant indicators were included in the multivariate analysis, and the best-fitting Cox risk regression model was constructed using likelihood ratio forward selection (Forward LR). Tumor type and tumor stage were considered as categorical variables. Multicollinearity was tested by linear regression analysis, and variance inflation factor (VIF) > 10 or tolerance < 0.1 was considered as collinearity existence. A new index body composition score (BCS) was constructed based on the results of multivariate Cox. The role of BCS in predicting prognosis was explored in all study participants and in different tumor type subgroups separately, and a time-dependent ROC curve of BCS was plotted. A nomogram was generated to develop the scored-CT system with statistically significant variables of multivariate Cox regression. C-index and calibration curve analyses were performed to validate the predictive accuracy of the scored-CT system.

### Ethics approval and consent to participate

The study was approved by the Ethics Committee of the first affiliated hospital of Jilin University (2017-362), and all the patient data in this research was approved by this committee. All study participants have filled out written informed consent for participation.

## Results

### Baseline characteristic

A total of 575 subjects meeting the criteria were finally enrolled in the study, including 362 males (63.0%) and 213 females (37.0%). The mean age of the overall study population was 59.63 years. Colorectal cancer dominated with 310 cases, accounting for 53.9%, while the rest were patients with gastric cancer, hepatocellular carcinoma, pancreatic cancer, esophageal cancer and biliary system malignant tumors in that order. The majority of patients were stage IV when diagnosed, accounting for 38.1% of the total study subjects (See Table 1). A total of 195 participants died during the follow-up.

**Table 1:** Baseline characteristics of enrolled participants.

Characteristics (N = 575)	N (%)/mean ± s.d
Age	59.63 ± 10.13
Sex	
Male	362 (63.0%)
Female	213 (37.0%)
Tumor type	
Gastric cancer	115 (20.0%)
Colorectal cancer	310 (53.9%)
Hepatocellular carcinoma	60 (10.4%)
Esophageal cancer	30 (5.2%)
Pancreatic cancer	36 (6.3%)
Biliary malignant tumor	24 (4.2%)
Stage	
I	41 (7.1%)
II	134 (23.3%)
III	181 (31.5%)
IV	219 (38.1%)
Smoke history (n = 471^a^)	
No	250
Yes/ever	221
Alcohol history (n = 471^a^)	
No	341
Yes/ever	130
Nutrition support (n = 471^a^)	
No	285
Yes/ever	186
BMI	22.55 ± 3.35
KPS	88.30 ± 11.30
Nutritional status^b^	
Well-nourished	148 (25.74%)
Moderate malnutrition	260 (45.22%)
Severe malnutrition	167 (29.04%)
HRQoL	68.70 ± 20.15
Treatment after admission	
Surgical resection	350 (60.87%)
Chemotherapy	497 (86.43%)
Radiotherapy	36 (6.26%)
Targeted therapy	50 (8.70%)
Immunotherapy	17 (2.96%)

BMI, body mass index; KPS, Karnofsky performance status; PG-SGA, the patient-generated subjective global assessment; HRQoL, health-related quality of life.

^a^Due to missing or low-quality data, some participants lack information on smoke history, alcohol history and nutritional support. The sample size that can be used after quality control is 471, accounting for 82% of the total sample size.

^b^Nutritional status was classified according to the PG-SGA scores: Well-nourished (PG-SGA scores ≤ 2 points); Moderate malnutrition (3 ≤ PG-SGA scores ≤ 8 points); Severe malnutrition (PG-SGA scores ≥ 9 points).

### Correlation analysis of study indicators

**Figure 2:**
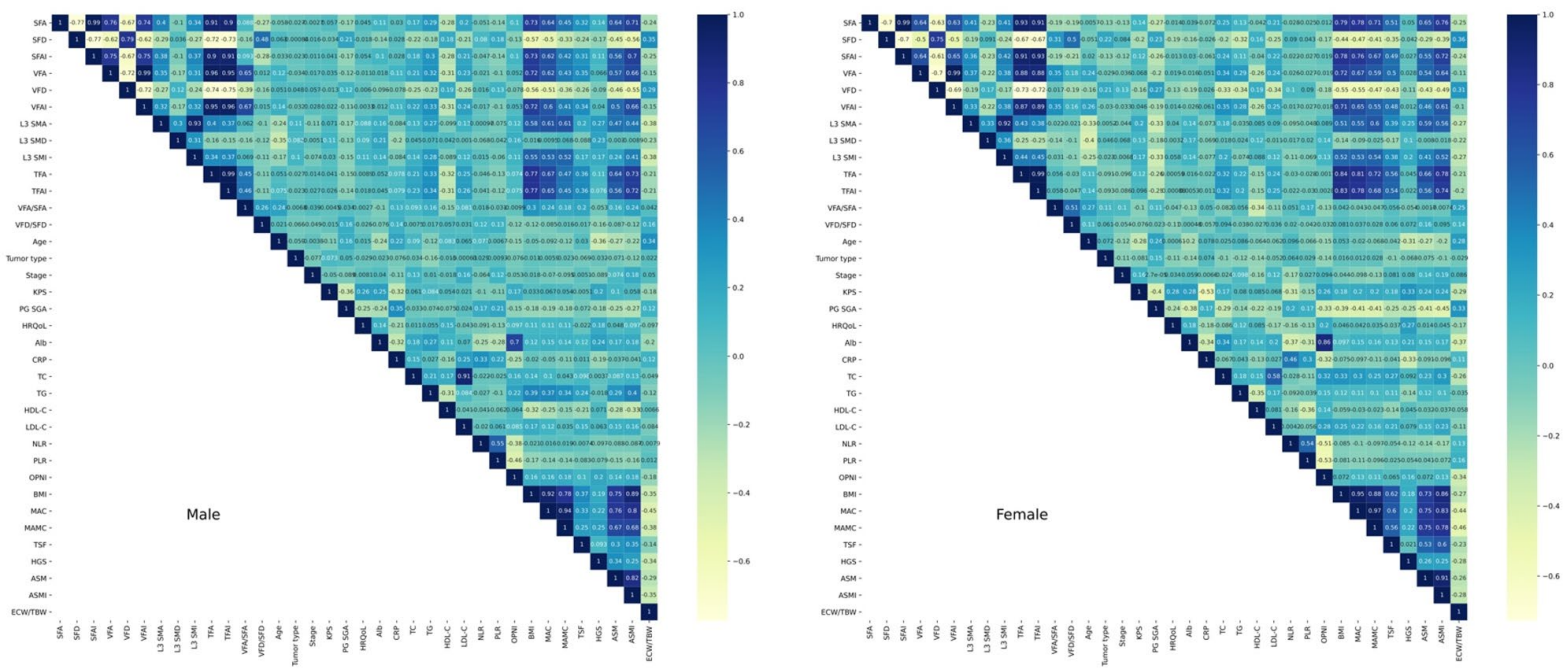
Correlation matrix separately for males and females between CT-related indicators and other variables.

In terms of gender, male and female patients were statistically different in several CT-related indicators (SFA: 98.87 ± 50.06 vs. 144.29 ± 65.98, *P* < 0.001; SFD: − 88.93 ± 11.30 vs. − 96.10 ± 9.75, *P* < 0.001; SFAI: 33.75 ± 16.87 vs. 58.20 ± 26.70, *P* < 0.001; VFA: 110.47 ± 72.22 vs. 87.19 ± 53.55, *P* < 0.001; L3 SMA: 139.55 ± 25.45 vs. 95.50 ± 16.96, *P* < 0.001; L3 SMD: 38.44 ± 7.10 vs. 32.66 ± 6.83, *P* < 0.001; L3 SMI: 47.87 ± 8.56 vs. 38.57 ± 6.30, *P* < 0.001; TFA: 208.69 ± 115.29 vs. 231.48 ± 108.49, *P* = 0.036; TFAI: 71.27 ± 39.11 vs. 93.40 ± 44.07, *P* < 0.001; VFA/SFA: 1.10 ± 0.50 vs. 0.65 ± 0.47, *P* < 0.001; VFD/SFD: 1.02 ± 0.09 vs. 0.95 ± 0.08, *P* < 0.001; Males vs. Females, respectively). We observed that although VFD and VFAI were higher in males than those in females, this difference was not statistically significant (See Supplemental Table 1). We then plotted the correlation matrix separately for male and female and could see that CT-related indicators were significantly correlated with several clinical information, anthropometric indicators, serological indicators and BIA indicators (See Fig. 2).

### Determination of prognosis-related indicators

The optimal cut-off values for CT-related indices were first determined by X-tile. We calculate the best cut-off values for males and females separately. The final results were obtained as follows: in males, SFA was 92.01 cm^2^, SFD was -91.61 HUs, SFAI was 28.51 cm^2^/m^2^, VFA was 69.20 cm^2^, VFD was − 87.37 HUs, VFAI was 20.72 cm^2^/m^2^, L3 SMA was 125.70 cm^2^, L3 SMD was 36.50HUs, L3 SMI was 43.11 cm^2^/m^2^, TFA was 296.78 cm^2^, TFAI was 79.53 cm^2^/m^2^, VFA/SFA was 0.69 and VFD/SFD was 1.10; in females, SFA was 65.96 cm^2^, SFD was − 86.45 HUs, SFAI was 26.65 cm^2^/m^2^, VFA was 27.06 cm^2^, VFD was − 81.11 HUs, VFAI was 10.70 cm^2^/m^2^, L3 SMA was 89.30 cm^2^, L3 SMD was 25.48 HUs, L3 SMI was 33.83 cm^2^/m^2^, TFA was 81.62 cm^2^, TFAI was 33.86 cm^2^/m^2^, VFA/SFA was 0.69 and VFD/SFD was 1.02 (See Supplemental Fig. 1). We divided the patients into high and low value groups according to the results of the corresponding indicators.

With OS as the observation endpoint, the results of the univariate Cox risk regression model revealed that several indicators correlated with OS (See Table 2). In a further multivariate Cox risk regression model, we fitted a Forward LR approach to form the best model, and the results suggested that among all CT-related indicators, only VFD, L3 SMI, and VFA/SFA were associated with prognosis of cancer patients (HR 2.473, 95% CI of HR 1.716–3.564, *P* < 0.001; HR 0.580, 95% CI of HR 0.385–0.872, *P* = 0.009; HR 1.812, 95% CI of HR 1.201–2.733, *P* = 0.005; See Table 2). In addition, tumor type, stage, surgical resection or not, KPS, PG-SGA and Alb were also associated with OS in cancer patients. Multicollinearity was tested by linear regression analysis, and VIF > 10 or tolerance < 0.1 was considered as collinearity existence. Supplemental Table 2 showed no collinearity existed among these variables, indicating that the multivariate Cox risk regression model by a Forward LR approach was well constructed.

**Table 2:** Univariate and multivariate Cox regression models for potential factors associated with overall survival in cancer patients.

Variables	Categorization	Univariate analysis		Multivariate analysis	
		HR (95% CI)	P	HR (95% CI)	P
Sex	Female	Reference			
	Male	0.989 (0.740–1.322)	0.940		
Age	< 60	Reference			
	≥ 60	1.298 (0.972–1.734)	0.077		
Tumor type					
	Gastric cancer	Reference		Reference	
	Colorectal cancer	0.571 (0.397–0.821)	0.003	0.820 (0.461–1.459)	0.499
	Hepatocellular carcinoma	0.740 (0.434–1.261)	0.268	1.836 (0.864–3.902)	0.114
	Esophageal cancer	1.396 (0.768–2.535)	0.274	1.973 (0.918–4.243)	0.082
	Pancreatic cancer	3.695 (2.269–6.017)	< 0.001	3.424 (1.613–7.270)	0.001
	Biliary malignant tumor	1.685 (0.911–3.117)	0.096	2.123 (0.936–4.816)	0.072
Stage					
	I	Reference		Reference	
	II	2.212 (0.769–6.360)	0.141	2.580 (0.855–7.783)	0.093
	III	3.793 (1.375–10.467)	0.010	3.677 (1.223–11.052)	0.020
	IV	10.315 (3.784–28.121)	< 0.001	7.153 (2.278–22.465)	0.001
Treatment after admission					
	Surgical resection	0.267 (0.199–0.357)	< 0.001	0.501 (0.307–0.819)	0.006
	Chemotherapy	1.699 (1.058–2.730)	0.028		
	Radiotherapy	1.648 (0.988–2.751)	0.056		
	Targeted therapy	1.505 (0.966–2.346)	0.071		
	Immunotherapy	1.016 (0.451–2.292)	0.969		
Smoke	No/never	Reference			
	Yes/ever	1.299 (0.937–1.802)	0.117		
Alcohol	No/never	Reference			
	Yes/ever	1.088 (0.762–1.555)	0.642		
KPS score		0.979 (0.968–0.990)	< 0.001	0.984 (0.969–1.000)	0.050
PG-SGA		1.087 (1.051–1.124)	< 0.001	1.065 (1.014–1.118)	0.011
HRQoL		0.989 (0.981–0.996)	0.004		
BMI	< 25	Reference			
	≥ 25	0.616 (0.423–0.896)	0.011		
Alb		0.950 (0.925–0.977)	< 0.001	0.957 (0.921–0.994)	0.023
CRP		1.004 (0.999–1.008)	0.123		
TC		0.954 (0.795–1.143)	0.608		
TG		0.783 (0.568–1.079)	0.135		
HDL-C		0.497 (0.240–1.031)	0.060		
LDL-C		1.216 (0.964–1.533)	0.099		
NLR		1.024 (0.997–1.052)	0.082		
PLR		1.002 (1.001–1.003)	< 0.001		
OPNI		0.960 (0.941–0.980)	< 0.001		
MAC		0.913 (0.872–0.956)	< 0.001		
MAMC		0.917 (0.870–0.967)	0.001		
HGS		0.982 (0.966–0.998)	0.028		
TSF		0.980 (0.955–1.006)	0.131		
ASM		0.988 (0.957–1.020)	0.454		
ASMI		0.964 (0.848–1.096)	0.575		
ECW/TBW	< 0.40	Reference			
	≥ 0.40	2.148 (1.495–3.087)	< 0.001		
SFA	Low	Reference			
	High	0.497 (0.358–0.690)	< 0.001		
SFD	Low	Reference			
	High	2.636 (1.872–3.712)	< 0.001		
SFAI	Low	Reference			
	High	0.459 (0.330–0.639)	< 0.001		
VFA	Low	Reference			
	High	0.621 (0.441–0.875)	0.007		
VFD	Low	Reference		Reference	
	High	2.722 (1.935–3.829)	< 0.001	2.473 (1.716–3.564)	< 0.001
VFAI	Low	Reference			
	High	0.594 (0.418–0.843)	0.004		
L3 SMA	Low	Reference			
	High	0.485 (0.365–0.645)	< 0.001		
L3 SMD	Low	Reference			
	High	0.820 (0.614–1.095)	0.178		
L3 SMI	Low	Reference		Reference	
	High	0.481 (0.359–0.644)	< 0.001	0.580 (0.385–0.872)	0.009
TFA	Low	Reference			
	High	0.597 (0.427–0.836)	0.003		
TFAI	Low	Reference			
	High	0.579 (0.417–0.803)	0.001		
VFA/SFA	Low	Reference		Reference	
	High	1.780 (1.225–2.587)	0.003	1.812 (1.201–2.733)	0.005
VFD/SFD	Low	Reference			
	High	1.881 (1.190–2.972)	0.007		

KPS, Karnofsky performance status; PG-SGA, the patient-generated subjective global assessment; HRQoL, health-related quality of life; BMI, body mass index; Alb, albumin; CRP, C-reactive protein; TC, serum total cholesterol; TG, serum triglycerides; HDL-C, serum high-density lipoprotein cholesterol; LDL-C, serum low-density lipoprotein cholesterol; NLR, neutrophil to lymphocyte ratio; PLR, platelet to lymphocyte ratio; OPNI, Onodera's prognostic nutritional index; MAC, circumference of the mid-upper arm; MAMC, mid-upper arm muscle circumference; HGS, grip strength of the non-dominant hand; TSF, skinfold thickness; ASM, appendicular skeletal muscle mass; ASMI, ASM index; ECW, extracellular water; TBW, total body water; SFA, subcutaneous fat area; SFD, subcutaneous fat density; SFAI, SFA index; VFA, visceral fat area; VFD, visceral fat density; VFAI, VFA index; L3 SMA, skeletal muscle area of the third lumbar vertebrae; L3 SMD, skeletal muscle density of third lumbar vertebrae; L3 SMI, skeletal muscle index of third lumbar vertebrae; TFA, total fat area; TFAI, TFA index.

### Establishment of body composition score based on CT

We combined VFD, L3 SMI and VFA/SFA to create a new CT-based index body composition score (BCS). According to the Cox results, we defined VFD-High, L3 SMI-Low, VFA/SFA-High as the risk group and assigned a score of 1. The rest were assigned a score of 0. BCS was the sum of the scores of each group (See Supplemental Table 3). A Venn diagram of patients with different CT characteristics was presented (See Supplemental Fig. 2).

**Table 3:** Univariate and multivariate Cox risk regression model on overall survivals of body composition score in study participants.

Variables	OS (Model 1)		OS (Model 2)		OS (Model 3)		OS (Model 4)	
	Crude HR (95% CI)	Crude P	Adjusted HR (95% CI)	Adjusted P	Adjusted HR (95% CI)	Adjusted P	Adjusted HR (95% CI)	Adjusted P
Overall	2.101 (1.721–2.565)	< 0.001	2.507 (2.000–3.142)	< 0.001	2.037 (1.647–2.519)	< 0.001	2.036 (1.644–2.521)	< 0.001
Sub-group by tumor type								
Gastric cancer	1.474 (0.915–2.375)	0.111	1.456 (0.828–2.559)	0.192	1.471 (0.788–2.747)	0.225	2.035 (0.783–5.290)	0.145
Colorectal cancer	2.339 (1.637–3.343)	< 0.001	2.900 (1.903–4.419)	< 0.001	2.699 (1.758–4.142)	< 0.001	2.693 (1.755–4.132)	< 0.001
Hepatocellular carcinoma	2.960 (1.553–5.643)	0.001	4.775 (2.242–10.172)	< 0.001	4.944 (2.485–9.836)	< 0.001	4.863 (2.451–9.649)	< 0.001
Esophageal cancer	2.061 (1.050–4.047)	0.036	2.360 (1.151–4.841)	0.019	2.980 (1.263–7.026)	0.013	4.431 (1.472–13.335)	0.008
Pancreatic cancer	2.040 (1.192–3.492)	0.009	2.807 (1.581–4.983)	< 0.001	2.807 (1.581–4.983)	< 0.001	1.905 (1.127–3.219)	0.016
Biliary system malignancies	1.577 (0.590–4.213)	0.363	4.073 (1.075–15.430)	0.039	5.016 (1.262–19.936)	0.022	23.829 (1.242–457.310)	0.035

Model 1: unadjusted; Model 2: adjusted for age, sex, stage, tumor type; Model 3: adjusted for age, sex, stage, tumor type, KPS score, PG-SGA, HRQoL; Model 4: adjusted for age, sex, stage, tumor type, KPS score, PG-SGA, HRQoL, Alb, PLR, OPNI, BMI, MAC, MAMC, HGS, ECW/TBW.

KPS, Karnofsky Performance Status; PG-SGA, the Patient-Generated Subjective Global Assessment; HRQoL, health-related quality of life; BMI, body mass index; Alb, albumin; PLR, platelet to lymphocyte ratio; OPNI, Onodera’s prognostic nutritional index; MAC, circumference of the mid-upper arm; MAMC, mid-upper arm muscle circumference; HGS, grip strength of the non-dominant hand; ECW, extracellular water; TBW, total body water.

To further clarify the value of BCS, we explored the role of BCS in predicting prognosis in the overall study population, as well as after grouping according to tumor type, respectively. To minimize clinical bias, four different adjustment models were constructed. Model 1: unadjusted; Model 2: adjusted for age, sex, stage, tumor type; Model 3: adjusted for age, sex, stage, tumor type, KPS score, PG-SGA, HRQoL; Model 4: adjusted for age, sex, stage, tumor type, KPS score, PG-SGA, HRQoL, Alb, PLR, OPNI, BMI, MAC, MAMC, HGS, ECW/TBW. In the overall study population, BCS was significantly associated with patient OS (Model 1: HR 2.101, 95% CI of HR 1.721–2.565, *P* < 0.001; Model 2: HR 2.507, 95% CI of HR 2.000–3.142, *P* < 0.001; Model 3: HR 2.037, 95% CI of HR 1.647–2.519, *P* < 0.001; Model 4: HR 2.036, 95% CI of HR 1.644–2.521, *P* < 0.001; See Table 3). Further subgroup analysis was done according to tumor types, and BCS was a valid prognostic predictor in colorectal cancer, hepatocellular carcinoma, esophageal cancer, pancreatic cancer and biliary system malignancies (HR 2.693, 95% CI of HR 1.755–4.132, *P* < 0.001; HR 4.863, 95% CI of HR 2.451–9.649, *P* < 0.001; HR 4.431, 95% CI of HR 1.472–13.335, *P* = 0.008; HR 1.905, 95% CI of HR 1.127–3.219, *P* = 0.016; HR 23.829, 95% CI of HR 1.242–457.310, *P* = 0.035, respectively; See Table 3). In the subgroup analysis of gastric cancer patients, after adjusting by models, we did not find BCS to be statistically significant in prognostic prediction. However, the HR remained greater than 1, representing a trend that higher BCS in patients with gastric cancer still has a poorer prognosis (HR 2.035, 95% CI of HR 0.783–5.290, *P* = 0.145).

### Time-dependent ROC curve of BCS

**Figure 3:**
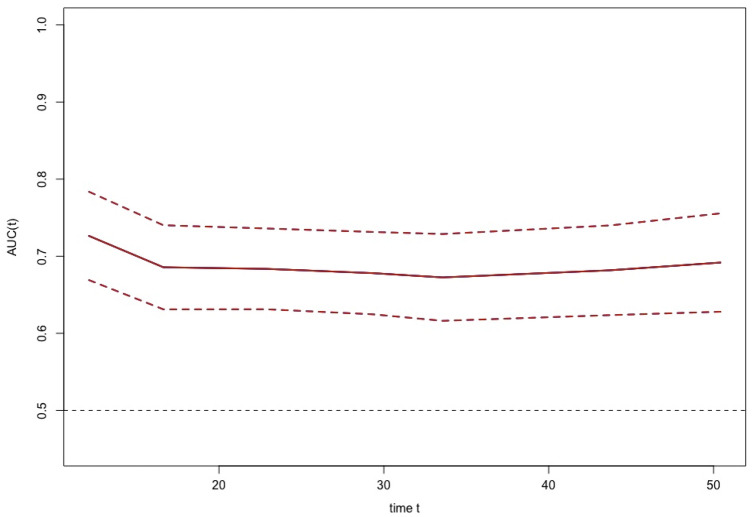
The time-dependent ROC curve of body composition score in predicting overall survivals. *In the graph, the horizontal coordinate time t is measured in months.

To test the predictive validity of the BCS in predicting the prognosis of patients with malignancy, we plotted the time-dependent ROC curve of the BCS. It was observed that the predictive validity of BCS remained around 0.70 throughout the follow-up period, indicating the high prognostic validity of BCS (See Fig. 3).

### Construction and validation of scored-CT system

**Figure 4:**
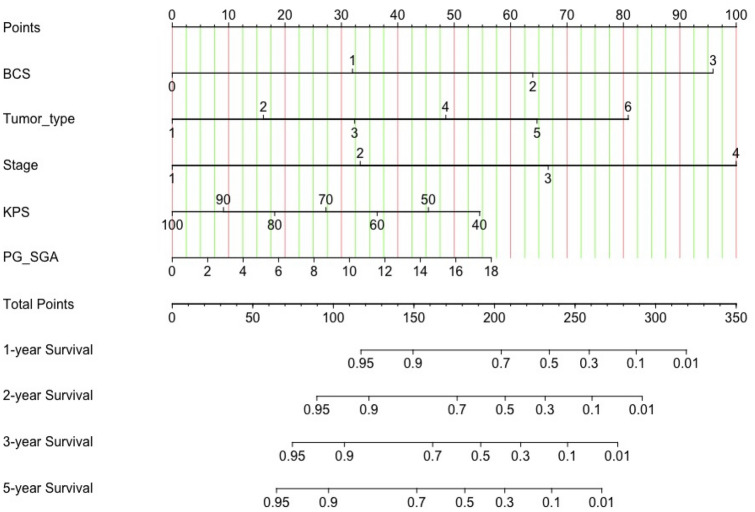
The scored-CT system by nomogram used to predict the clinical outcome of patients with digestive system malignancy. Tumor type: 1 = Gastric cancer; 2 = Colorectal cancer; 3 = Hepatocellular carcinoma; 4 = Esophageal cancer; 5 = Pancreatic cancer; 6 = Biliary malignant tumor. Stage: 1 = I; 2 = II; 3 = III; 4 = IV.

**Figure 5:**
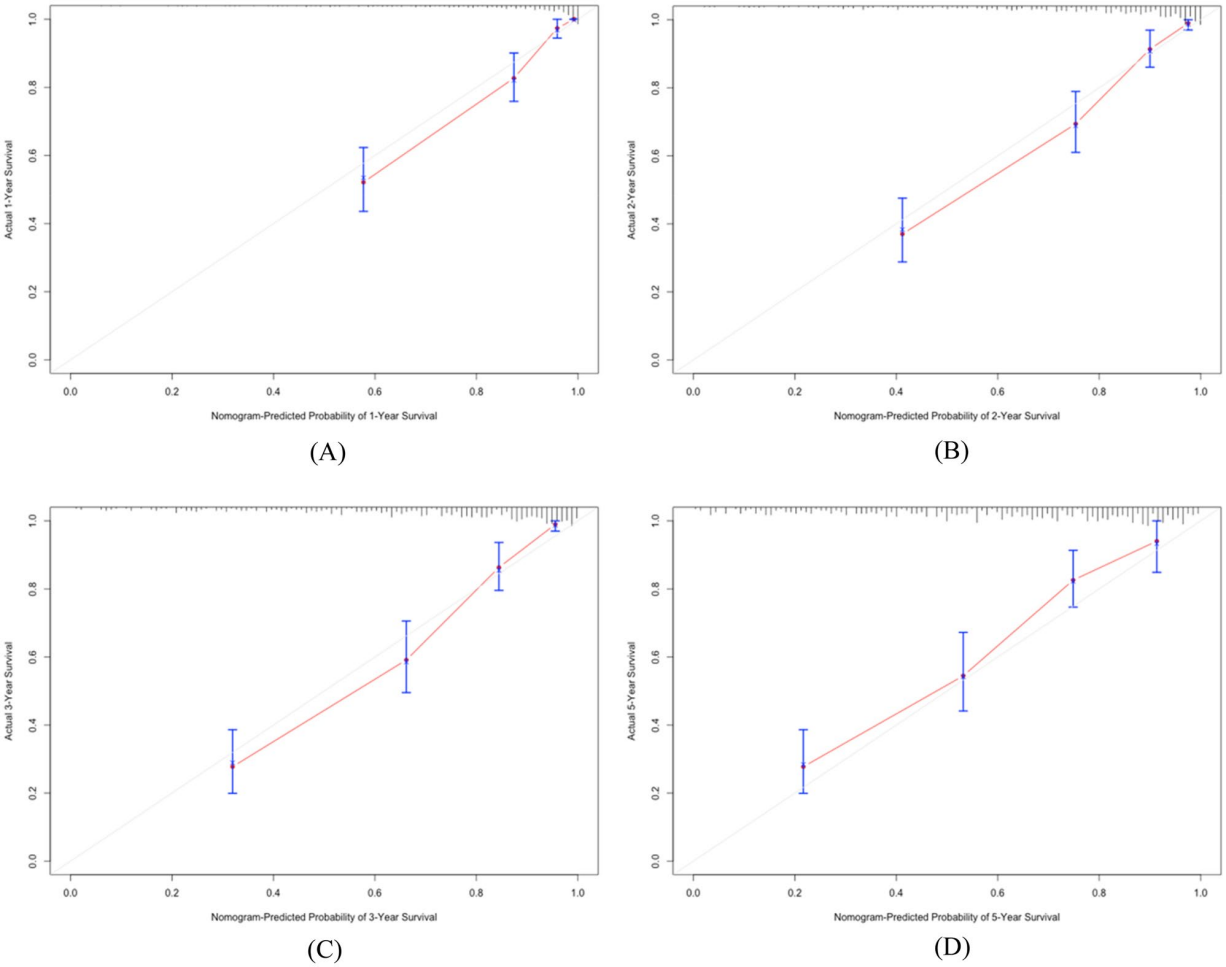
Calibration curves of study participants for the nomogram predictions of the 1-, 2-, 3-and 5-year overall survival. (**A**) Calibration curves of nomogram predictions of the 1-year overall survival; (**B**) Calibration curves of nomogram predictions of the 2-year overall survival; (**C**) Calibration curves of nomogram predictions of the 3-year overall survival; (**D**) Calibration curves of nomogram predictions of the 5-year overall survival.

A nomogram was constructed according to tumor type, stage, KPS, PG-SGA and BCS to develop a scored-CT system (See Fig. 4). Each subtype within these variables was assigned a score based on a point scale. Each variable site was located on the axis, and then a line was drawn straight upward to the Points axis to determine how many points the patient received from the variables. A scored-CT system to determine the estimated probability of survival at each time point was easily obtained by adding the total score and locating it on the total point scale. To clarify the reliability of the model, we calculated the C-index of the model as 0.813 (95% CI 0.797–0.829). The calibration curves were then plotted for 1, 2, 3, and 5 years (See Fig. 5). It can be seen that the model we constructed predicts the survival rate at 1, 2, 3, and 5 years in high agreement with the actual survival rate of the patients.

## Discussion

The changes in the body composition of patients with malignant tumors are often dramatic. The prognosis of different individuals in the same disease state or tumor burden condition varies greatly due to differences in body composition^19,20^. This means that "precision therapy" for human components can help patients achieve better treatment benefits. In this study, we focused on the skeletal muscle and fat status of patients with malignant tumors because they represent the level of individual energy reserves and metabolic status^21–23^, which are of great value to the human body.

We focused on digestive malignancies because abdominal CT is an essential diagnostic and evaluation method for digestive malignancies, which facilitated the acquisition of CT data without increasing the risk of radiation exposure to patients. Skeletal muscle mass at the third lumbar level is significantly associated with the prognosis of various diseases including malignant tumors, which has been confirmed in previous studies^11,24,25^. Therefore, relevant CT indicators at the third lumbar level are still selected as the object of exploration in this study. It can be seen from the results of the correlation matrix that CT-related indicators are correlated with a variety of biological indicators including PLR, which means that data indicators extracted from CT images have the potential to be used as prognostic biomarkers for cancer patients. We also observed that although BMI was correlated with fat distribution VFA/SFA, the correlation was very low. We believe that this is precisely due to the defects of BMI in the assessment of body composition, which cannot truly reflect the situation of each component and also makes it difficult to detect latent muscle loss in obese patients. Further Cox regression results showed that VFD, L3 SMI and VFA/SFA were CT-related indicators that could significantly affect OS of cancer patients.

It can be seen that for patients with digestive system malignancies, L3 SMI can be used as an important indicator to evaluate their OS. There is a tendency for skeletal muscle mass and function to decline with age, particularly in patients with co-morbid cancers, as prolonged energy depletion results in the forced breakdown of multiple body components involved in metabolism, including increased proteolysis, decreased amino acid transport and regeneration^26^. When the functional status and quality of skeletal muscle are severely impaired in some patients to a degree that we call sarcopenia, this is associated with poorer clinical outcomes because patients with combined sarcopenia face a greater risk of systemic therapeutic drug toxicity. As we know, platinum-based chemotherapeutic agents are mainly distributed in skeletal muscle tissue for metabolism, and current chemotherapy drug regimens mainly use a body surface area based approach to drug dosing, which may result in high dose levels of systemic chemotherapy drugs given in obese patients with low skeletal muscle mass, causing systemic chemotherapy-related toxicity^27^. In addition, cancer patients with a combination of sarcopenia are more likely to have postoperative complications and consequently have significantly shorter progression free survivals and OS^28–30^. This could explain the poorer disease prognosis and shorter overall survival time for the L3 SMI-Low patients in our study.

While we acknowledge the value of skeletal muscle in disease and its important role in disease progression, the severe reduction in muscle mass is easily masked by the phenotype of obesity^22^, potentially misleading clinical decision-makers in their subsequent therapeutic management strategies. Obesity is associated with cardiovascular risk, diabetes, and other diseases, even promoting the development of malignancies and accelerating tumor progression^31^. However, as we explore obesity more deeply, we find that not all obesity risks are consistent, and the concept of metabolic healthy obesity (MHO) is proposed. Although increased energy storage in the form of triglycerides in the abdominal region, especially in the visceral region, promotes an unhealthy metabolic phenotype, energy storage in the form of triglycerides in subcutaneous adipose tissue of the buttocks and legs is considered beneficial for metabolic health^32^. So, the distribution of fat directly affects the metabolic status of the whole body. Compared to metabolic unhealthy obesity (MUO), patients with MHO have higher cardiorespiratory fitness and physical activity, higher insulin sensitivity, lower levels of inflammatory markers and normal adipose tissue function. Although the risk of combined cardiovascular disease is lower in MHO patients than that in MUO patients, it is still higher than that in metabolically healthy lean individuals^21,33^. Therefore, MHO may be a temporary phenotype of MUO, and with further lipid accumulation adipose tissue dysfunction occurs, eventually developing into MUO. Unhealthy fat distribution is a "breeding ground" for many diseases, including cardiovascular disease and cancer^34–37^. Because of this, it is not only important to pay attention to changes in the amount of total body fat, but also the distribution of fat.

In our study, we found that patients with VFA/SFA-High had significantly worse prognosis and shorter OS, so we believe that visceral fatty obesity is significantly associated with poor prognosis in patients with digestive system malignancies. Similarly, we also observed that patients with VFD-High had a worse prognosis, again suggesting that the accumulation of abdominal visceral fat promotes the development of cancer. Similarly, adipose distribution promotes the metastasis and further aggressive development of tumor cells to a certain extent, which is related to the fact that adipose accumulation promotes the production of chemokines and also serves as a sufficient energy source for tumor cell proliferation^38^. Adipose tissue can be described as an endocrine organ distributed throughout the body, but subcutaneous fat is mostly metabolically inactive, whereas visceral adipose tissue is more endocrinologically active. Excessive fat deposition increases adipokine release and leads to adipocyte hypoxia, allowing for increased cytokines and immune cell recruitment, which may lead to higher levels of systemic inflammation^21,39^. We hypothesize that the level of immune function in cancer patients differs significantly due to the distribution of adipose tissue, which in turn leads to individual differences in response to subsequent treatment and prognosis.

As can be seen from the above, not only skeletal muscle is important for cancer patients, but also fat distribution is an important indicator of prognosis. To better predict the survival of individual patients in clinical work, we combined the muscle and fat conditions of patients with cancer to construct a new BCS index in our study, based on which to reflect both muscle and fat. The time-dependent ROC curve showed that the predictive validity of BCS is stable around 0.70 during the whole follow-up period, which means that our new index BCS was of great predictive validity for patients with gastrointestinal malignancies and is expected to become a new tumor biomarker. We then constructed a prognostic prediction model called the scored-CT system, using the BCS, combined with tumor type, tumor stage, KPS score, and PG-SGA score, which has excellent predictive validity for both short-term survival prognosis and long-term 5-year survival in patients with digestive system malignancies. We did not include Alb in the scored-CT system because it would made clinical data more difficult to obtain, while the improvement in predictive power was not significant.

As determined by the current study, body composition affects the outcome of patients with malignant tumors in many ways, and the main theory is that skeletal muscle is the "buffer" of anti-tumor therapy. Skeletal muscle content is not only related to the toxicity after chemotherapy, but also may be related to the efficacy of chemotherapy, the response to targeted therapy and even the response to immunotherapy^40,41^. This reflects the interaction among nutrition, inflammation and immunity. Malnutrition is widespread in patients with malignant tumors, and about 25% to 80% of cancer patients have experienced varying degrees of malnutrition^42,43^. The incidence of malnutrition is significantly higher in patients with digestive system tumors because the tumor location often leads to more serious eating obstruction and dysfunction of nutrient absorption. From an objective perspective, malnutrition not only affects the choice of anti-tumor treatment, reduces response rates to treatment, increases subjective symptoms, and lowers the quality of life, but also increases the incidence of complications and mortality^44,45^. Therefore, it is necessary to adopt individualized nutritional therapy for cancer patients, which greatly improves clinical outcomes. For malnourished patients, physicians can improve PG-SGA scores through parenteral and enteral nutrition to anticipate a better clinical outcome. For patients with a high risk of death such as VFA/SFA-High and L3 SMI-Low, we can optimize fat distribution and improve skeletal muscle index by dose resistance training, reasonably controlling diet and moderate physical activity^46–49^, to control BCS at a lower level and achieve better survival. In short, our study achieved the extraction of the corresponding indicators from CT images, which successfully constructed a new biomarker, BCS, to effectively predict the survival time of patients with digestive system malignant tumors. On this basis, the scored-CT system successfully combined CT indicators, tumor disease status and nutritional status to intervene in the disease process from these three aspects to optimize clinical outcomes.

To some extent, our study comprehensively reflects the overall situation of cancer patients in China, as this is a real-world clinical study in an Asian population with OS, with prognosis as the observation endpoint. We believe that it has the appropriate guidance value for clinical application. Compared with previous studies, our BCS index and scored-CT system comprehensively reflected the interaction of muscle and fat, with corresponding advantages. The corresponding limitation of this study is that our study is a retrospective study, it is difficult to avoid the existence of partial data bias. Secondly, we lack the corresponding external validation data, which may lead to certain shortcomings of this prediction model. In addition, the prediction model in this research was constructed by patients with malignant tumors of the digestive system, which encompasses several tumor types. Due to the sample size limitation, it is difficult to build an accurate prediction model for each type. However, based on the continuous improvement of Investigation on Nutrition Status and its Clinical Outcome of Common Cancers (INSCOC) database construction, we also expect further external data validation in the subsequent research.

## Conclusion

In summary, our study acknowledges the value of the role of muscle and adipose tissue in patients with digestive malignancies, and clarifies that CT-based body composition analysis can be used for prognostic prediction. In addition, the prognostic prediction model we constructed has good predictive value and was of good stability. This is a prediction model that both muscle and adipose tissue are used in the construction as proposed for Asian patients. We expect that this model will be applied in clinical practice to provide guidance for cancer patients.

### Supplementary Information

Supplementary Figure 1.

Supplementary Figure 2.

Supplementary Table 1.

Supplementary Table 2.

Supplementary Table 3.

## Data Availability

Materials described in the manuscript, including all relevant raw data, will be freely available to any scientist wishing to use them for non-commercial purposes, without breaching participant confidentiality. Any investigator interested in viewing raw data may contact us by email: zhengkaiwen1995@163.com.
