# Supplementary material for: CT-based muscle and adipose measurements predict prognosis in patients with digestive system malignancy
Source: Sci Rep. 2024 Jun 6;14:13036. doi: 10.1038/s41598-024-63806-1 (PMC11156914; doi:10.1038/s41598-024-63806-1)
Supplement: Supplementary file 1 — Supplementary Figure 1. [file 41598_2024_63806_MOESM1_ESM.docx]

Supplemental Figure 1. Optimal cutoff values of CT-related indicators based on overall survivals calculated by X-tile software.


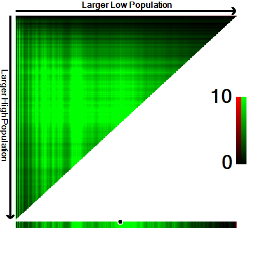

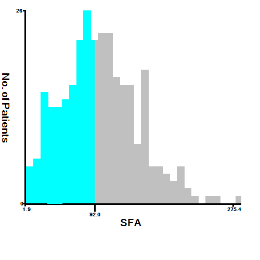

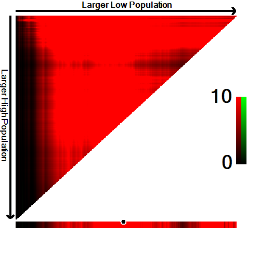

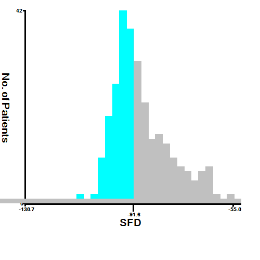

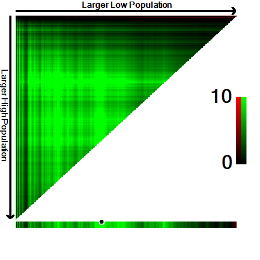

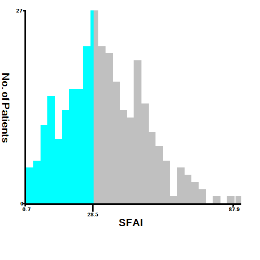

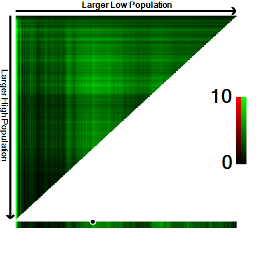

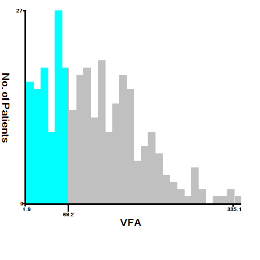

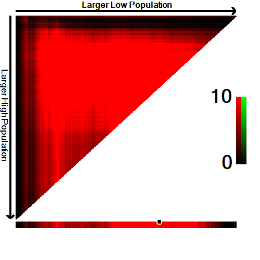

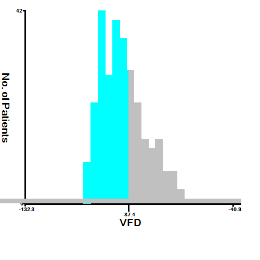

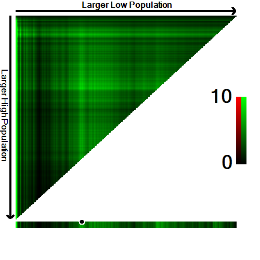

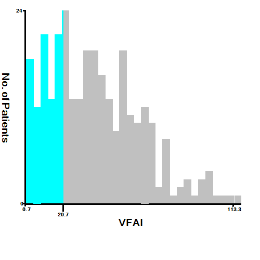

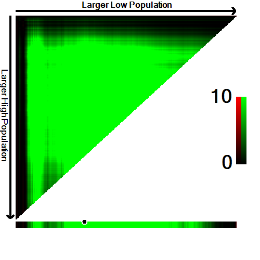

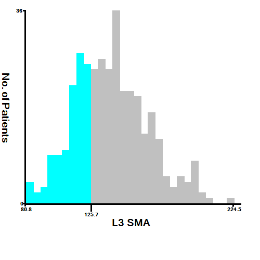

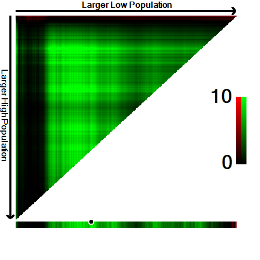

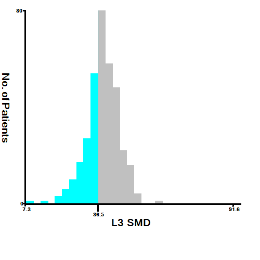

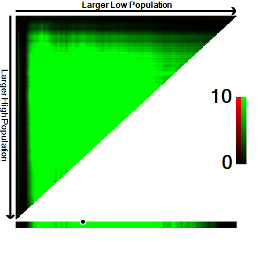

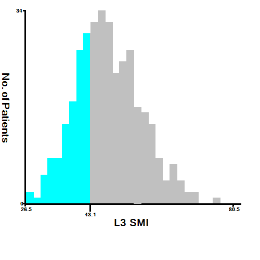

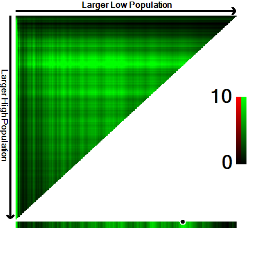

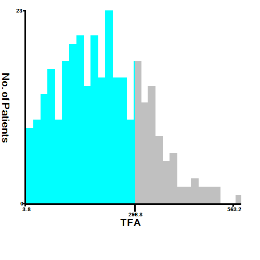

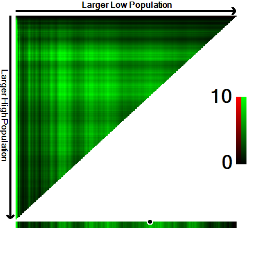

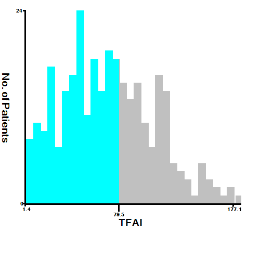

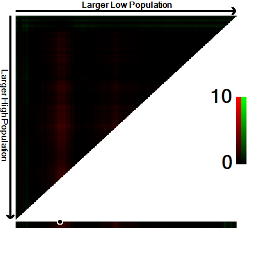

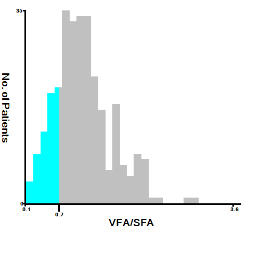

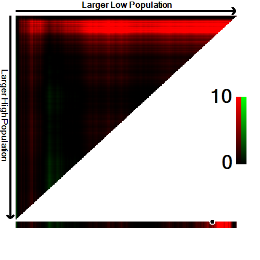

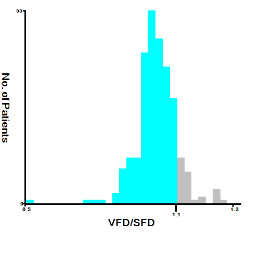


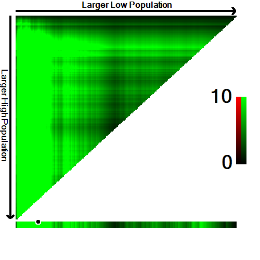

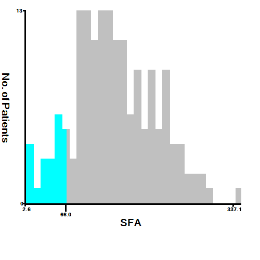

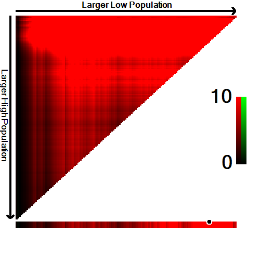

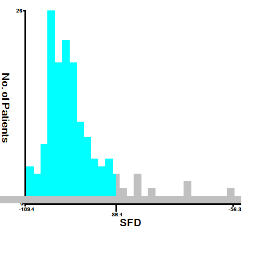

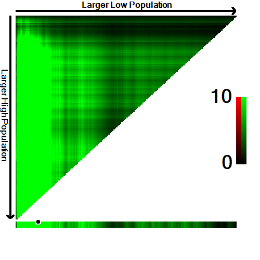

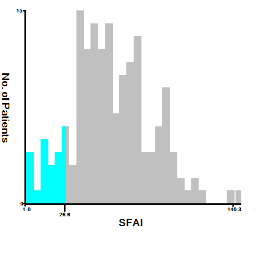

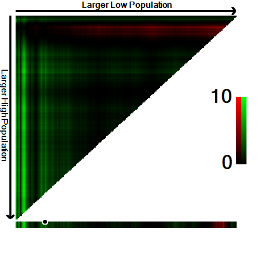

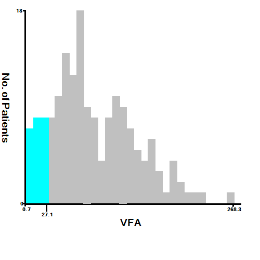

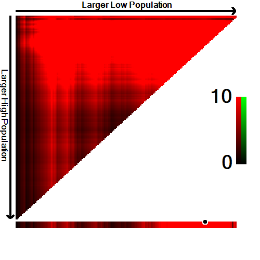

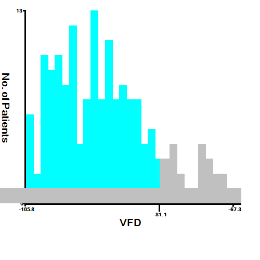

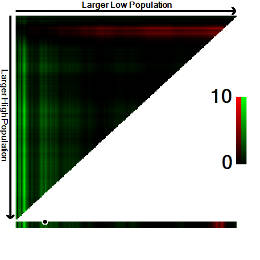

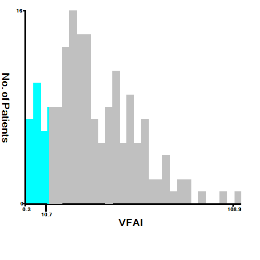

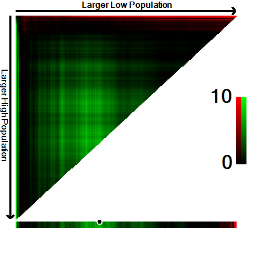

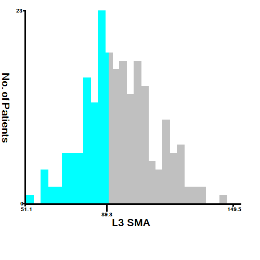

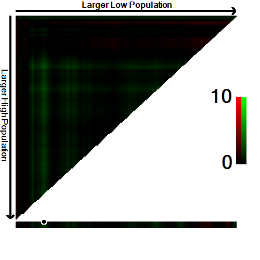

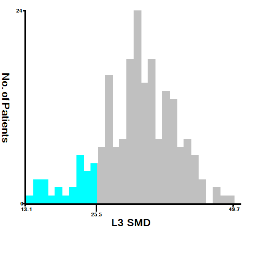

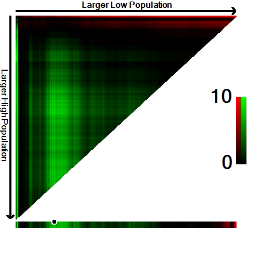

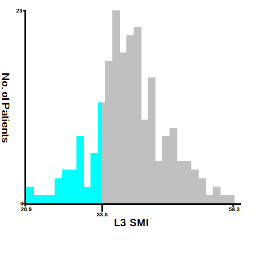

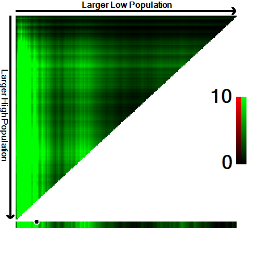

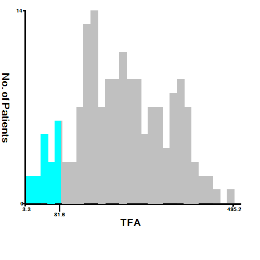

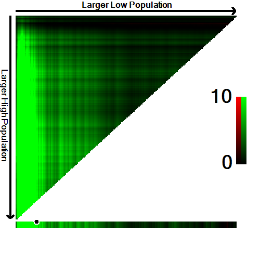

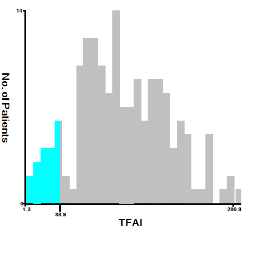

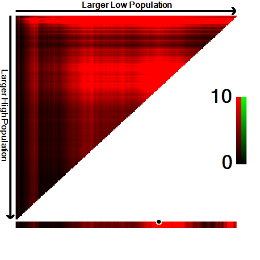

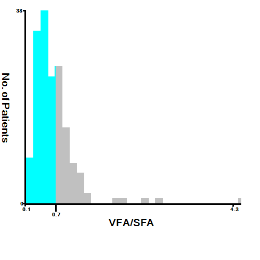

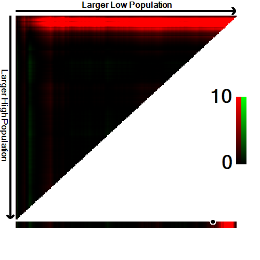

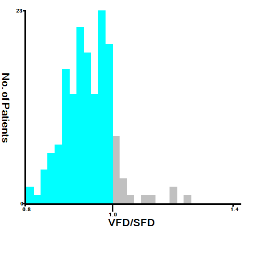


Note: The optimal cutoff values were as follows; For male, SFA was 92.01 cm^2^, SFD was -91.61 HUs, SFAI was 28.51 cm^2^/m^2^, VFA was 69.20 cm^2^, VFD was -87.37 HUs, VFAI was 20.72 cm^2^/m^2^, L3 SMA was 125.70 cm^2^, L3 SMD was 36.50 HUs, L3 SMI was 43.11 cm^2^/m^2^, TFA was 296.78 cm^2^, TFAI was 79.53 cm^2^/m^2^, VFA/SFA was 0.69 and VFD/SFD was 1.10; For female, SFA was 65.96 cm^2^, SFD was -86.45 HUs, SFAI was 26.65 cm^2^/m^2^, VFA was 27.06 cm^2^, VFD was -81.11 HUs, VFAI was 10.70 cm^2^/m^2^, L3 SMA was 89.30 cm^2^, L3 SMD was 25.48 HUs, L3 SMI was 33.83 cm^2^/m^2^, TFA was 81.62 cm^2^, TFAI was 33.86 cm^2^/m^2^, VFA/SFA was 0.69 and VFD/SFD was 1.02.
