# Supplementary figures and images for: CT-based muscle and adipose measurements predict prognosis in patients with digestive system malignancy
Source: Sci Rep. 2024 Jun 6;14:13036. doi: 10.1038/s41598-024-63806-1 (PMC11156914; doi:10.1038/s41598-024-63806-1)

Supplemental Figure 2. Venn diagram of patients with different CT features.


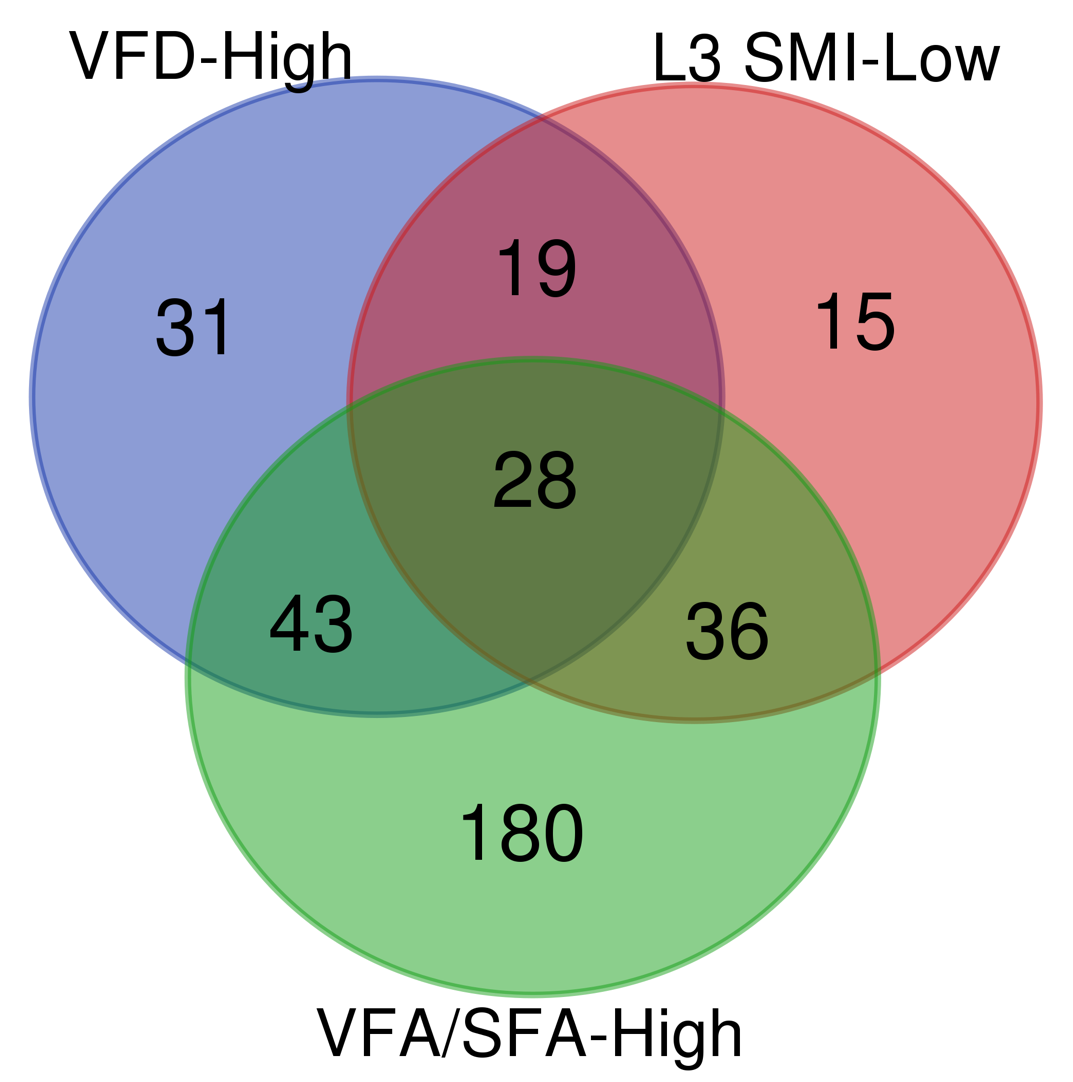

Supplement: Supplementary file 2 — Supplementary Figure 2. [file 41598_2024_63806_MOESM2_ESM.docx]
