# Supplementary material for: CT-based muscle and adipose measurements predict prognosis in patients with digestive system malignancy
Source: Sci Rep. 2024 Jun 6;14:13036. doi: 10.1038/s41598-024-63806-1 (PMC11156914; doi:10.1038/s41598-024-63806-1)
Supplement: Supplementary file 3 — Supplementary Table 1. [file 41598_2024_63806_MOESM3_ESM.docx]

Supplemental Table 1. Characteristic of body composition between male and female among study participants.

| **Characteristic** | **mean­­±s.d.** | | **t** | **P** |
| --- | --- | --- | --- | --- |
|  | Male | Female |  |  |
| SFA (cm^2^) | 98.87­­±50.06 | 144.29­­±65.98 | -7.778 | <0.001 |
| SFD (HUs) | -88.93­­±11.30 | -96.10­­±9.75 | 7.054 | <0.001 |
| SFAI (cm^2^/m^2^) | 33.75±16.87 | 58.20±26.70 | -10.758 | <0.001 |
| VFA (cm^2^) | 110.47­­±72.22 | 87.19­­±53.55 | 3.974 | <0.001 |
| VFD (HUs) | -89.85­­±10.55 | -91.35­­±9.04 | 1.510 | 0.132 |
| VFAI (cm^2^/m^2^) | 37.73±24.70 | 35.20±21.80 | 1.115 | 0.266 |
| L3 SMA (cm^2^) | 139.55­­±25.45 | 95.50­­±16.96 | 24.795 | <0.001 |
| L3 SMD (HUs) | 38.44­­±7.10 | 32.66­­±6.83 | 9.390 | <0.001 |
| L3 SMI (cm^2^/m^2^) | 47.87­­±8.56 | 38.57­­±6.30 | 14.911 | <0.001 |
| TFA (cm^2^) | 208.69­­±115.29 | 231.48­­±108.49 | -2.101 | 0.036 |
| TFAI (cm^2^/m^2^) | 71.27±39.11 | 93.40±44.07 | -5.625 | <0.001 |
| VFA/SFA | 1.10­­±0.50 | 0.65­­±0.47 | 9.714 | <0.001 |
| VFD/SFD | 1.02­­±0.09 | 0.95­­±0.08 | 7.412 | <0.001 |
| ASM (kg) | 22.44­­±2.55 | 14.90­­±2.38 | 35.057 | <0.001 |
| ASMI (kg/m^2^) | 7.69­­±0.67 | 5.98­­±0.79 | 26.363 | <0.001 |
| MAC (cm) | 29.87±2.88 | 28.00±3.18 | 7.232 | <0.001 |
| MAMC (cm) | 27.01±2.29 | 24.04±2.25 | 15.128 | <0.001 |
| HGS (kg) | 30.39±9.13 | 18.01±6.98 | 16.481 | <0.001 |
| TSF (mm) | 15.89±8.61 | 17.92±6.43 | -2.670 | 0.008 |
| BMI (kg/m^2^) | 22.68­­±3.17 | 22.33­­±3.64 | 1.156 | 0.248 |
| ECW/TBW | 0.3865­­±0.0104 | 0.3900­­±0.0099 | -3.846 | <0.001 |

SFA, subcutaneous fat area; SFD, subcutaneous fat density; SFAI, SFA index; VFA, visceral fat area; VFD, visceral fat density; VFAI, VFA index; L3 SMA, skeletal muscle area of the third lumbar vertebrae; L3 SMD, skeletal muscle density of third lumbar vertebrae; L3 SMI, skeletal muscle index of third lumbar vertebrae; TFA, total fat area; TFAI, TFA index; ASM, appendicular skeletal muscle mass; ASMI, ASM index; MAC, circumference of the mid-upper arm; MAMC, mid-upper arm muscle circumference; HGS, grip strength of the non-dominant hand; TSF, skinfold thickness; BMI, body mass index; ECW, extracellular water; TBW, total body water.
