# Supplementary material for: CT-based muscle and adipose measurements predict prognosis in patients with digestive system malignancy
Source: Sci Rep. 2024 Jun 6;14:13036. doi: 10.1038/s41598-024-63806-1 (PMC11156914; doi:10.1038/s41598-024-63806-1)
Supplement: Supplementary file 4 — Supplementary Table 2. [file 41598_2024_63806_MOESM4_ESM.docx]

Supplemental Table 2. Multicollinearity test of influencing factors by linear regression analysis.

| **Factor** | **Tolerance** | **Variance inflation factor (VIF)** |
| --- | --- | --- |
| Sex | 0.295 | 3.389 |
| Age | 0.694 | 1.441 |
| Tumor type | 0.982 | 1.078 |
| Stage | 0.924 | 1.082 |
| KPS | 0.730 | 1.370 |
| PG-SGA | 0.671 | 1.490 |
| HRQoL | 0.822 | 1.217 |
| BMI | 0.273 | 3.662 |
| Alb | 0.289 | 3.459 |
| PLR | 0.669 | 1.495 |
| OPNI | 0.262 | 3.820 |
| MAC | 0.164 | 6.087 |
| MAMC | 0.271 | 3.691 |
| HGS | 0.517 | 1.935 |
| ECW/TBW≥0.4 | 0.777 | 1.287 |
| SFA | 0.180 | 5.570 |
| SFD | 0.340 | 2.942 |
| SFAI | 0.172 | 5.829 |
| VFA | 0.112 | 8.898 |
| VFD | 0.497 | 2.014 |
| VFAI | 0.123 | 8.157 |
| L3 SMA | 0.432 | 2.317 |
| L3 SMD | 0.733 | 1.364 |
| L3 SMI | 0.466 | 2.147 |
| TFA | 0.234 | 4.265 |
| TFAI | 0.230 | 4.332 |
| VFA/SFA | 0.549 | 1.821 |
| VFD/SFD | 0.762 | 1.313 |

Note: VIF >10 or tolerance < 0.1 was considered as collinearity existence.
