# Supplementary material for: CT-based muscle and adipose measurements predict prognosis in patients with digestive system malignancy
Source: Sci Rep. 2024 Jun 6;14:13036. doi: 10.1038/s41598-024-63806-1 (PMC11156914; doi:10.1038/s41598-024-63806-1)
Supplement: Supplementary file 5 — Supplementary Table 3. [file 41598_2024_63806_MOESM5_ESM.docx]

Supplemental Table 3. Body composition score calculated by CT features.

| **Categorization** | **Scores of each sub-group** | | |
| --- | --- | --- | --- |
|  | VFD | L3 SMI | VFA/SFA |
| Low | 0 | 1 | 0 |
| High | 1 | 0 | 1 |

***Body composition score (BCS)=VFD+L3 SMI+VFA/SFA**

The cut-off values were determined by X-tile. VFD: -87.37 HUs vs. -81.11 HUs (Male vs. Female); L3 SMI: 43.11 cm^2^/m^2^ vs. 33.83 cm^2^/m^2^ (Male vs. Female); VFA/SFA: 0.69 vs. 0.69 (Male vs. Female).
